# The polymorphisms of miRNA‐binding site in *MLH3* and *ERCC1* were linked to the risk of colorectal cancer in a case–control study

**DOI:** 10.1002/cam4.1319

**Published:** 2018-03-08

**Authors:** Qianye Zhang, Xiao Zheng, Xiaoxia Li, Deyu Sun, Ping Xue, Guopei Zhang, Mingyang Xiao, Yuan Cai, Cuihong Jin, Jinghua Yang, Shengwen Wu, Xiaobo Lu

**Affiliations:** ^1^ Department of Toxicology School of Public Health China Medical University Shenyang China; ^2^ Department of Colorectal Surgery The Fourth Affiliated Hospital China Medical University Shenyang China; ^3^ Department of Radiation Oncology(6) (Digestive system cancer) Cancer Hospital of China Medical University Shenyang China

**Keywords:** Colorectal Cancer, *ERCC1*, miR‐193a‐3p, *MLH3*, polymorphism on 3′UTR

## Abstract

Colorectal cancer (CRC), as a malignant tumor of lower digestive tract, has been found to have an increasing morbidity and mortality in China. It was particularly important to find some earlier biomarkers to predict the risk and prognosis. In this study, several polymorphisms on 3′UTR of three DNA repair genes including *MLH3* rs10862, *ERCC1* rs3212986, *ERCC1* rs735482, *ERCC1* rs2336219, and *OGG1* rs1052133 were chosen by bioinformatics exploration, and then, a case–control study of 200 CRC cases and controls was performed. Furthermore, a dual‐luciferase assay was also carried out to certify whether the candidate miRNA can regulate its target gene and the selected SNPs have a valid effect on the target miRNA. Finally, both of *ERCC1* rs3212986 and *MLH3* rs108621 were shown to be associated with the risk of CRC. Comparing with rs3212986 CC genotype, AA was at a higher risk (OR = 3.079, 95% CI: 1.192–7.952). For *MLH3* rs108621, comparing with TT genotype, CC and TC were at a higher risk of CRC in male (OR = 5.171, 95% CI: 1.009–26.494; OR = 1.904, 95% CI: 1.049–3.455). Interestingly, an analysis combining both *ERCC1* rs3212986 and *MLH3* rs108621 also showed an increased risk of CRC. In addition, a dual‐luciferase assay showed that miR‐193a‐3p could regulate *MLH3,* and the polymorphism rs108621 could alter the miR‐193a‐3p binding to *MLH3*. Therefore, *MLH3* rs108621 may be associated with the risk of CRC due to the effect of miR‐193a‐3p on *MLH3,* which reminded the possibility as potential susceptibility biomarkers to predict the risk of CRC.

## Introduction

Colorectal carcinoma (CRC), as one of the most common malignancies, is the third common cancer and the fourth leading cause of cancer death worldwide 1. Modern medicine prevalently considered that the occurrence and development of CRC were attributed to the interaction of environment and genetic factors 2. Diagnosis and treatment at the earlier stage of CRC contribute to increase the survival rate and life quality of patient 3. Therefore, it is vital to search for some valid biomarkers to predict the risk and prognosis of CRC.

Chemical carcinogens can damage DNA, leading to mutation and canceration. DNA repair systems in normal cell can repair those damages to maintain genomic stability. However, the individual DNA repair capacity is different to human body or cells. Generally speaking, at least four DNA repair systems such as base excision repair (BER), nucleotide excision repair (NER), mismatch repair (MMR), and double‐strand break repair (DSBR) are involved in DNA repair in human 4. The genetic polymorphisms in DNA repair pathways were reported to be linked with the occurrence and development of CRC 5, 6, 7. For example, A allele in Gly39Glu (116G > A) polymorphism of *MutS Homolog 6* (*MSH6*) gene in MMR pathway was found to increase the risk of the sporadic CRC in a Polish population 8. Thr241Met (rs861539) polymorphism of *X‐ray Repair Cross‐Complementing 3* (*XRCC3*) in DSBR pathway might be regarded as a potential molecular marker of CRC 9. *X‐ray Repair Cross‐Complementing 1* (*XRCC1*) plays an important role in BER, and A allele in its polymorphism Arg399Gln (rs25487) can decrease the risk to develop CRC 10.

3′‐Untranslated region (3′UTR) of eukaryotic mRNA is a momentous functional element, which plays a critical role in cellular location and regulates gene expression 11, which has an effect on regulation of noncoding RNA especially microRNA (miRNA) by binding its specific site 12. As we have known, the ectopic expression of miRNAs may be closely related to many complex diseases as tumors. For example, miR‐21 could downregulate (human MutS Homolog 2) hMSH2 and (human MutS Homolog 6) hMSH6 protein expression, which can alter the DNA repair capacity of MMR 13. Similarly, miR‐31‐5p disturbed the cell cycle by binding 3′UTR of *MutL Homolog 1* (*MLH1*) and contributed to the development of cancers 14. The 3′UTR polymorphism may influence single or multiple gene expression, playing a critical role in cellular phenotype, proliferation, and differentiation. Some related CRC studies of miRNA and its binding site polymorphism have been gradually paid attention. T allele of rs61764370 located in *KRAS proto‐oncogene* (*KRAS*) 3′UTR was found to increase the risk of CRC 15. *Nucleotide Binding Oligomerization Domain Containing 2* (*NOD2*), an inflammation gene, and its 3′UTR polymorphism rs3135500 were shown a significant relationship with the risk of CRC 16. However, the SNPs rs17468 and rs2317676 on 3′UTR of *Integrin Subunit Beta 1* (*ITGB1*) were shown to have an effect on increasing the risk of CRC 17.

To date, there are few researches to study the relationship between the SNPs in 3′UTR of DNA repair genes and the susceptibility to CRC. Our current study selected several DNA repair genes in three DNA repair pathways including NER, BER, MMR systems as candidate genes and predicted the effect of SNPs of miRNA‐binding sites on its relevant miRNA by bioinformatics exploration. Further, we performed a case–control study to evaluate the association between the selected SNPs and the risk of CRC. Lastly, a luciferase report in vitro experiment was also carried out to certify whether the candidate miRNA can regulate its target gene and the selected SNPs have a valid effect on its target miRNA. Therefore, this study will provide a platform to find some valuable evidence to support the possibility of polymorphisms as potential susceptibility biomarkers in predicting the risk of CRC.

## Material and Methods

### Study subjects

200 CRC patients were collected from the Fourth Affiliated Hospital of China Medical University in Shenyang (a city in the northeast of China) from October 2013 to July 2015. The inclusion criteria of cases were definite diagnosis of primary CRC based on standard clinical and histological criteria. The Institutional Review Board of China Medical University approved the study and informed consent was obtained from all participants prior to the study. All activities involving human subjects were done under full compliance with government policies and the Helsinki Declaration. After the study, procedures were explained and all questions were answered, and subjects signed informed consent forms. Demographic data were obtained using a questionnaire. Two hundred healthy control subjects matched for age and sex with the CRC cases were recruited from a Physical Examination Center of the First Affiliated Hospital of China Medical University. Venous blood (2 mL) was drawn from each subject and incubated with sodium citrate anticoagulation.

### Bioinformatics screening

Search the keywords: “colorectal cancer,” “NER,” “BER,” “MMR,” and “Susceptibility” on GeneCards (http://www.genecards.org/). Three public available Web sites were used to search for the SNPs on 3′UTR of the above candidate genes. Minor allele frequency (MAF) of those SNPs in Chinese Han population was found in Hapmap (http://hapmap.ncbi.nlm.nih.gov/), NCBI (https://www.ncbi.nlm.nih.gov/), UCSC (https://genome.ucsc.edu/). According to MAF and sample size, determine the candidate SNPs. Use Targetscan (http://www.targetscan.org/) and PolymiRTsto (http://compbio.uthsc.edu/miRSNP/) forecast the miRNAs binding the candidate SNPs. The relevance between the miRNAs and the risk of CRC was explored by literature mining.

### TaqMan^®^ SNP genotyping assays

2 mL of venous blood was drawn from each subject and collected with a folic acid sodium anticoagulant. DNA was routinely extracted by phenol–chloroform extraction.

*ERCC1* (rs3212986, C > A, assay ID is C_2532948_10, part number is 4351379; rs735482, A > C, assay ID is C_341729_10, part number is 4351379; rs2336219, G > A, assay ID is C_16204465_10, part number is 4351379), *MLH3* (rs108621, T > C, assay ID is C_2178406_10, part number is 4351379), *OGG1*(rs1052133, G>C, assay ID is C_3095552_1_, part number is 4351379) was purchased from ABI Company(ABI, US, Stagapore) and analyzed by TaqMan^®^ sequencing on Roche 480 Real‐time PCR system (Roche, Basel, Switzerland). All PCR reagents were purchased from Roche Company (Roche).

The PCRs were performed in a 20 *μ*L reaction mixture: 10 *μ*L of probe Mix, 5 *μ*L (1x) of each probe and primer and 2 *μ*L of DNA (25 ng/*μ*L). The PCR included an initial step at 95°C for 10 min; 40 cycles of denaturation at 95°C for 10 sec, extension at 60°C for 1 min and 72°C for 1 sec; at the last, cool at 40°C for 30 sec.

### Dual‐luciferase reporter assay

Human embryonic kidney 293T (HEK‐293T) cells were purchased from the Cell Bank of the Shanghai Institute of Biochemistry and Cell Biology, Chinese Academy of Sciences, and cultured in Dulbecco's modified Eagle's medium (Hyclone, Logan, Utah, USA). The cells were supplemented with 10% fetal bovine serum (Hyclone) and maintained at 37°C, 5% CO_2_ in a humidified incubator.

The wild‐type (WT) 3′UTRs of *MLH3* containing putative miR‐193a‐3p and miR‐338‐3p binding sites were isolated by PCR using the primer pair the wild‐type (WT) forward: TAACAGAGAGAACCGGCCAGTATGCTGGC. The mutation‐type (MT): TAACAGAGAGAACCT‐CGAGTATACGAAA. The SNP‐type (SNP): TAACAGAGAGAACCGGCCAGTGTGCTGGC. WT, MT, and SNP *MLH3* 3′UTRs were cloned into pMIR‐REPORT vector containing a synthetic firefly luciferase gene which was specifically designed to be an intraplasmid transfection normalization reporter (Ohio, China). All of the constructed vectors were verified by sequencing.

HEK‐293T cells were cultured in 96‐well plates and cotransfected with 50 nmol/L of miR‐193a mimic or miR‐338‐3p mimic (miRNA mimic control), 50 ng of luciferase reporter vector, and 10 ng of pRL‐CMV Renilla luciferase reporter vector using Lipofectamine 3000 (Firefly: Renilla: Lipofectamine 3000 = 0.1 *μ*g:0.01 *μ*g:0.25 *μ*L). Forty‐eight hours after transfection, the luciferase activities were assayed using a luciferase assay kit. All experiments were performed four times in triplicate. For each sample, the firefly luciferase activity was normalized to the Renilla luciferase activity used as a control to standardize for the transfect efficiency.

### Statistical analysis

All data obtained were analyzed by SPSS 19.0 and GraphPad Prism 5.0 Software. A Fisher's exact test or chi‐squared (*χ*2) test was selected to compare the frequencies of the different genetic polymorphisms between the cases and controls. The chi‐square test was also used to evaluate the association between genetic polymorphisms and the risk of CRC. Multiple unconditional logistic regression analysis was used to estimate the odds ratios (ORs) and 95% confidence intervals (CIs) for high DNA adduct levels in participants with different haplotypes of the research genes. Two‐tailed *P *<* *0.05 was considered statistically significant.

## Results

### Candidate SNPs selection

The genes belonging to BER, NER, MMR, and its relevance scoring with CRC were searched through the Gene Cards Web site (see Table S1, S2, and S3). According to the purpose of the present study and the relevance scoring related to CRC, three DNA repair genes including *Excision Repair Cross‐Complementation Group 1* (*ERCC1*)*, 8‐Oxoguanine DNA Glycosylase* (*OGG1*), and *mutL homolog 3* (*MLH3*) were chosen as the candidate target genes. *ERCC1, OGG1,* and *MLH3* as three rate‐limiting enzyme genes in NER, BER, and MMR pathways were evaluated to be of value in predicting the risk of CRC.

Several SNPs in 3′UTR of *ERCC1*,* MLH3,* and *OGG1* were searched by the Web site of Targetscan and NCBI. According to the MAF of Chinese Han population of Beijing and the sample size of the present study, five SNPs in 3′UTR of *ERCC1*,* MLH3,* and *OGG1* were selected as the candidate SNPs (see Table 1).

**Table 1:** Alleles and MAF of the candidate SNPs

Gene	SNP	Genetic variation	Minor allele frequency (MAF)
*ERCC1*	rs735482	A/C	0.378
*ERCC1*	rs2336219	A/G	0.378
*ERCC1*	rs3212986	G/T	0.329
*MLH3*	rs108621	C/T	0.232
*OGG1*	rs1052133	C/G	0.45

### Characteristics of study population

200 CRC cases and 200 healthy controls matched by age and sex were collected in the present study. No significant differences were found between cases and controls in gender and age. The mean ages of cases and controls were 61.6 and 62.2 years old, respectively (see Table 2). The age distribution is in a skewed shape. The age range of 200 cases is from 22 to 83 years old. The 41–70 age group was found to have a larger proportion in cases, and its constituent ratio is 75.5%.

**Table 2:** Demographic characteristics of the patients and control individuals

Mean SD or *N* (%)			
Characteristics	Cases (*n* = 200)	Controls (*n* = 200)	*P* value
Age(year)	62.18 ± 12.637	61.59 ± 13.153	0.717
Male	62.35 ± 12.937	61.22 ± 13.821	0.666
Female	61.97 ± 13.322	62.06 ± 12.330	0.984
Sex			1.000
Male	111 (55.5)	111 (55.5)	
Female	89 (44.5)	89 (44.5)	

### Association analysis between the candidate SNPs and CRC risk

Hardy–Weinberg analysis of the candidate SNPs: To ensure those SNPs accord with the Hardy–Weinberg equilibrium, we tested the linkage disequilibrium (LD) for those SNPs. The result showed the entire candidate SNPs accorded with Hardy–Weinberg equilibrium.

Our data showed the result of the association analysis between the risk of CRC and the SNPs in Table 3. From the result, we found *ERCC1* rs3212986 was shown to be related to the risk of CRC: AA genotype had a higher risk than CC genotype (OR = 2.530, 95% CI: 1.144–5.597).

**Table 3:** Association between the candidate SNPs and CRC risk

SNPs	Cases	Controls	OR	*P*
	*N* (%)	*N* (%)	(95% CI)	
*MLH3* rs108621				0.494
TT	124 (62.0)	132 (66.0)	1.000	
TC	62 (31.0)	59 (29.5)	1.119 (0.726–1.724)	0.659
CC	14 (7.0)	9 (4.5)	1.656 (0.692–3.963)	0.283
TC+CC	76 (38.0)	68 (34.0)	1.190 (0.791–1.791)	0.466
*ERCC1* rs3212986				0.061
CC	100 (50.0)	115 (57.5)		
CA	78 (39.0)	75 (37.5)	1.196 (0.790–1.811)	0.459
AA	22 (11.0)	10 (5.0)	**2.530 (1.144**–**5.597)**	**0.023**
CA+AA	100 (50.0)	85 (42.5)	1.353 (0.912–2.007)	0.160
*ERCC1* rs735482				0.829
AA	51 (25.5)	47 (23.5)	1.000	
AC	107 (53.5)	113 (56.5)	0.873 (0.542–1.405)	0.628
CC	42 (21.0)	40 (20.0)	0.968 (0.538–1.740)	1.000
AC+CC	149 (73.5)	153 (76.5)	0.897 (0.569–1.416)	0.727
*ERCC1* rs2336219				0.676
GG	55 (27.5)	48 (24.0)	1.000	
GA	104 (52.0)	112 (56.0)	0.810 (0.506–1.297)	0.404
AA	41 (20.5)	40 (20.0)	0.895 (0.499–1.602)	0.708
GA + AA	145 (72.0)	152 (76.0)	0.833 (0.531–1.304)	0.493
*OGG1* rs1052133				0.259
GG	82 (41.0)	73 (36.5)	1.000	
GC	82 (41.0)	98 (49.0)	0.745 (0.484–1.146)	0.190
CC	36 (18.0)	29 (14.5)	1.105 (0.618–1.978)	0.736
GC+CC	118 (59.0)	127 (63.5)	0.827 (0.553–1.237)	0.412

Bold values mean *P*<0.05.

### Stratified analysis by gender

To exclude the influence of gender, a stratified analysis was performed in this study. The associations between the SNPs and the risk of CRC in male population are shown in Table 4. In male, C allele of *MLH3* rs108621 was found to be related to a higher risk of CRC. Similarly, *ERCC1* rs3212986 was related to the susceptibility to CRC as well: AA genotype with more susceptible than CC genotype (OR = 4.043, 95% CI: 1.261–12.968). While in female, *MLH3* rs108621 C allele was found to link with a reduced risk of CRC.

**Table 4:** The association between the candidate SNPs and CRC risk, stratified by gender

Groups	Male		OR (95% CI)	*P*	Female		OR (95% CI)	*P*
	Cases (%)	Controls (%)			Cases (%)	Controls (%)		
*MLH3* rs108621				**0.004**				0.078
TT	57 (51.4)	79 (71.2)	1.000		67 (75.3)	53 (59.6)	1.000	
TC	45 (40.5)	30 (27.0)	**2.079 (1.171**–**3.691)**	**0.014**	17 (19.1)	29 (32.6)	**0.464 (0.231**–**0.933)**	**0.037**
CC	9 (8.1)	2 (1.8)	**6.237 (1.298**–**29.966)**	**0.013**	5 (5.6)	7 (7.9)	0.565 (1.170–1.881)	0.378
TC+CC	54 (48.6)	32 (28.8)	**2.339 (1.344**–**4.071)**	**0.004**	22 (24.7)	36 (40.4)	**0.483 (0.255**–**0.918)**	**0.037**
*ERCC1* rs3212986				0.045				0.637
CC	58 (52.3)	67(60.4)	1.000		42 (47.2)	48 (53.9)	1.000	
CA	39 (35.1)	40(36.0)	1.126 (0.641–1.980)	0.774	39 (43.8)	35 (39.3)	1.273 (0.688–2.358)	0.530
AA	14 (12.6)	4(3.6)	**4.043 (1.261**–**12.968)**	**0.021**	8 (9.0)	6 (6.7)	1.524 (0.489–4.749)	0.570
CA+AA	53 (47.7)	44 (39.6)	1.391 (0.817–2.369)	0.279	47 (52.8)	41 (46.1)	1.310 (0.727–2.361)	0.454
*ERCC1* rs735482				0.943				0.770
AA	27 (24.3)	27 (24.3)	1.000		24 (27.0)	20 (22.5)	1.000	
AC	59 (53.2)	61 (55.0)	0.967 (0.509–1.839)	1.000	48 (53.9)	52 (58.4)	0.769 (0.378–1.567)	0.588
CC	25 (22.5)	23 (20.7)	1.087 (0.499–2.366)	0.846	17 (19.1)	17 (19.1)	0.833 (0.340–2.043)	0.820
AC+CC	84 (75.7)	84 (75.7)	1.000 (0.542–1.846)	1.000	65 (73.0)	69 (77.5)	0.785 (0.396–1.555)	0.603
*ERCC1* rs2336219				0.716				0.874
GG	31 (27.9)	27 (24.3)	1.000		24(27.0)	21 (23.6)	1.000	
GA	55 (49.5)	61 (55.0)	0.785 (0.418–1.477)	0.521	49 (55.1)	51 (57.3)	0.841 (0.415–1.701)	0.720
AA	25 (22.5)	23 (20.7)	0.947 (0.440–2.037)	1.000	16 (18.0)	17 (19.1)	0.824 (0.335–2.024)	0.819
GA+AA	80 (72.1)	84 (75.7)	0.829 (0.455–1.511)	0.647	65 (73.0)	68 (76.4)	0.836 (0.425–1.646)	0.730
*OGG1* rs1052133				0.771				0.238
GG	44 (39.6)	42 (37.8)	1.000		38 (42.7)	31 (34.8)	1.000	
GC	43 (38.7)	48 (43.2)	0.855 (0.474–1.543)	0.653	39 (43.8)	50 (56.2)	0.636 (0.338–1.198)	0.199
CC	24 (21.6)	21 (18.9)	1.091 (0.530–2.246)	0.855	12 (13.5)	8 (9.0)	1.224 (0.445–3.368)	0.800
GC+CC	67 (60.4)	69 (62.2)	0.927 (0.540–1.591)	0.890	51 (57.3)	58 (65.2)	0.717 (0.392–1.314)	0.356

Bold values mean *P*<0.05.

### Stratified analysis by age

The associations between the candidate SNPs and the risk of CRC in the age <50 and the age ≥50 population are shown in Table 5. The results reminded that no SNPs were found to be related to the risk of CRC in the age <50 population, while *ERCC1* rs3212986 was found to have a linkage in the age ≥50 population. AA genotype has a higher risk of CRC than CC genotype in the polymorphism (OR = 4.106, 95% CI: 1.407–11.980).

**Table 5:** The association between the candidate SNPs and CRC risk, stratified by age

Groups	Age < 50		OR (95% CI)	*P*	Age ≥ 50		OR (95% CI)	*P*
	Cases (%)	Controls (%)			Cases (%)	Controls (%)		
*MLH3* rs108621				0.320				0.663
TT	20 (69.0)	22 (71.0)	1.000		104 (60.8)	110 (65.1)	1.000	
TC	7 (24.1)	9 (29.0)	0.856 (0.269–2.725)	1.000	55 (32.2)	50 (29.6)	1.163 (0.729–1.857)	0.553
CC	2 (6.9)	0 (0)	0.476 (0.347–0.654)	0.488	12 (7.0)	9 (5.3)	1.410 (0.571–3.486)	0.499
TC+CC	9 (31.0)	9 (29.0)	1.100 (0.364–3.320)	1.000	67 (39.2)	59 (34.9)	1.201 (0.773–1.866)	0.433
*ERCC1* rs3212986				0.744				**0.030**
CC	14 (48.3)	17 (54.8)	1.000		86 (50.3)	98 (58.0)	1.000	
CA	13 (44.8)	11 (35.5)	1.435 (0.492–4.184)	0.591	65 (38.0)	64 (37.9)	1.157 (0.738–1.816)	0.566
AA	2 (6.9)	3 (9.7)	0.810 (0.118–5.544)	1.000	20 (11.7)	7 (4.1)	**3.256 (1.313**–**8.073)**	**0.012**
CA+AA	15 (51.7)	14 (45.2)	1.301 (0.471–3.591)	0.796	85 (49.7)	71 (42.0)	1.364 (0.889–2.093)	0.159
*ERCC1* rs735482				0.951				0.805
AA	6 (20.7)	6 (19.4)	1.000		45 (26.3)	41 (24.3)	1.000	
AC	20 (69.0)	21 (66.7)	0.952 (0.263–3.448)	1.000	87 (50.9)	92 (54.4)	0.862 (0.515–1.442)	0.601
CC	3 (10.3)	4 (12.9)	0.750 (0.115–4.898)	1.000	39 (22.8)	36 (21.3)	0.987 (0.531–1.835)	1.000
AC+CC	23 (79.3)	25 (80.6)	0.920 (0.260–3.261)	1.000	126 (73.7)	128 (75.7)	0.897 (0.550–1.463)	0.709
*ERCC1* rs2336219				0.743				0.793
GG	8 (27.6)	6 (19.4)	1.000		47 (27.5)	42 (24.9)	1.000	
GA	18 (62.1)	21 (66.7)	0.643 (0.188–2.203)	0.544	86 (50.3)	91 (53.8)	0.845 (0.507–1.406)	0.603
AA	3 (10.3)	4 (12.9)	0.563 (0.090–3.518)	0.659	38 (22.2)	36 (21.3)	0.943 (0.509–1.749)	0.876
GA+AA	21 (72.4)	25 (80.6)	0.630 (0.188–2.107)	0.547	124 (72.5)	127 (75.1)	0.873 (0.538–1.416)	0.623
*OGG1* rs1052133				0.357				0.145
GG	10 (34.5)	16 (51.6)	1.000		72 (42.1)	57 (33.7)	1.000	
GC	11 (37.9)	10 (32.3)	1.760 (0.549–5.643)	0.388	71 (41.5)	88 (52.1)	0.639 (0.400–1.019)	0.075
CC	8 (27.6)	5 (16.1)	2.560 (0.652–10.059)	0.196	28 (16.4)	24 (14.2)	0.924 (0.484–1.763)	0.869
GC+CC	19 (65.5)	15 (48.4)	2.027 (0.716–5.736)	0.203	99 (57.9)	112 (66.3)	0.700 (0.451–1.087)	0.119

Bold values mean *P*<0.05.

### The synergistic effect of *MLH3* Rs108621 C and *ERCC1* Rs3212986 A alleles

Combining the stratified analysis, *MLH3* rs108621 C allele and *ERCC1* rs3212986 A allele were found to increase the risk of CRC especially in male population. Whether the synergistic effect of *MLH3* rs108621 C and *ERCC1* rs3212986 A alleles existed is an interesting question. If we donated *MLH3* rs108621 C allele and *ERCC1* rs3212986 A allele as the “harmful alleles,” we can group the data according to the number of carrying the “harmful alleles” and evaluate the risk of CRC (see Table 6). The result showed that people who carry 3 “harmful alleles” had a higher risk of CRC than those who carrying less “harmful alleles.” Therefore, the synergistic effect of *MLH3* rs108621 C allele and *ERCC1* rs3212986 A allele was considered as a more powerful biomarker to predict the risk of CRC.

**Table 6:** The association between the number of *MLH3* rs108621 C and *ERCC1* rs3212986 A alleles and CRC risk in male population (OR(95% CI))

Harmful alleles	0	1	2	3	4
0					
1	1.391 (0.755–2.562)				
2	1.920 (0.881–4.188)	1.380 (0.651–2.929)			
3	**1.355 (1.131**–**1.622)** a	**1.244 (1.093**–**1.416)** a	**1.500 (1.178**–**1.909)** a		
4	1.065 (0.976–1.161)	1.044 (0.983–1.109)	1.091 (0.967–1.231)	–	

a *P *<* *0.01.

Bold values mean *P*<0.05.

### Bioinformatics prediction of the MiRNAs and their binding sites

Both *ERCC1* rs735482 and *MLH3* rs108621 were shown to be associated with the risk of CRC in the present study. However, *MLH3* rs108621 was selected as a significant polymorphism to predict the risk of CRC due to the closed relationship between DNA repair pathway MMR and CRC. Furthermore, the miRNAs binding on the candidate SNP were also forecasted by Targetscan and PolymiRTs (See Fig. S1 & Table S4). Finally, miR‐338‐3p and miR‐193a were predicted to bind to *MLH3* rs108621.

**Figure 1:**
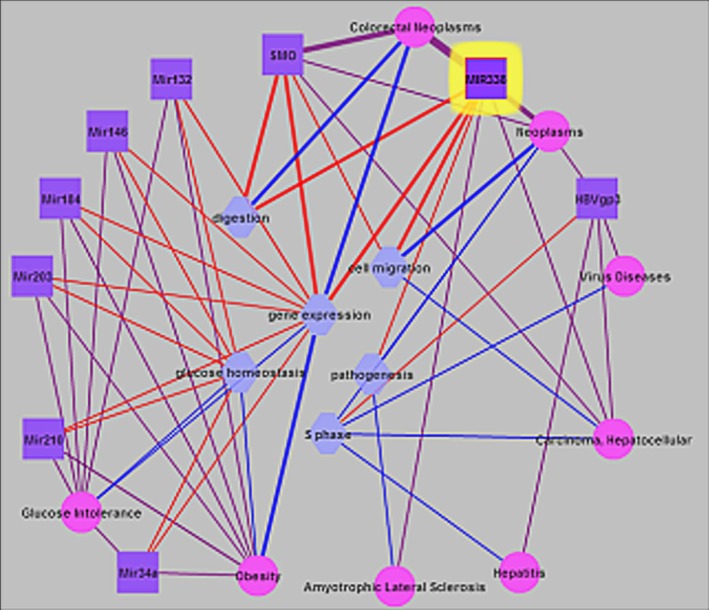
Literature mining of miR‐388‐3p.

To verify the association between the two miRNAs and CRC, literature mining of miR‐338‐3p and miR‐193a was carried out in the present study. Literature references of miR‐338‐3p showed that there were 57 related publications. The relevant diseases, the biological processes involved in, and its regulated genes to miR‐338‐3p have been mapped in Palm‐ist analysis software (Fig. 1). A close relationship between miR‐338‐3p and cancers especially CRC were shown. For the biological processes involved, miR‐338‐3p was also found to have some effects on cell migration and food digest. For the biological processes involved in, miR‐338‐3p may play an important role in the occurrence and development of CRC through *MLH3* regulation.

**Figure 2:**
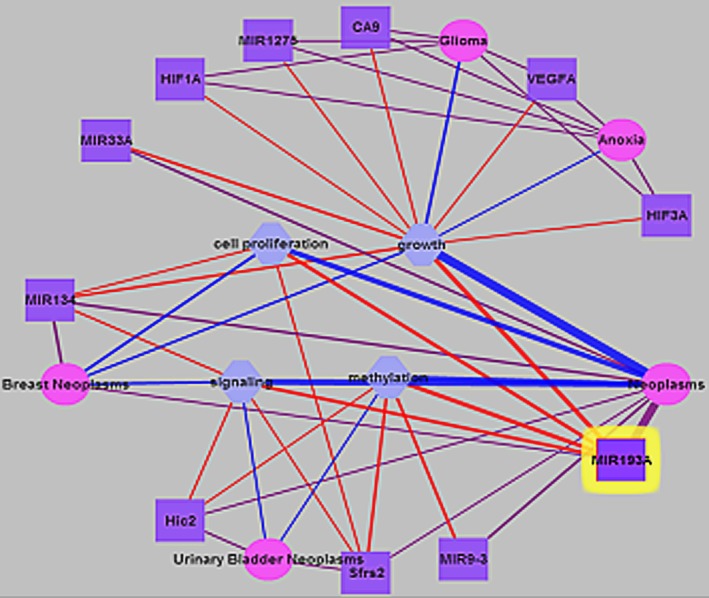
Literature mining of miR‐193a.

Literature references of miR‐193a showed that there were 94 literatures about miR‐193a (Fig. 2). After analysis, we found that miR‐193a is related to the occurrence and development of cancer. MiR‐193a involves in multiple biological process such as cell proliferation, growth, and methylation of tumorigenesis. Those evidences reminded miR‐193a can play a valuable role in the development of cancers.

### Luciferase reporter assay in vitro

**Figure 3:**
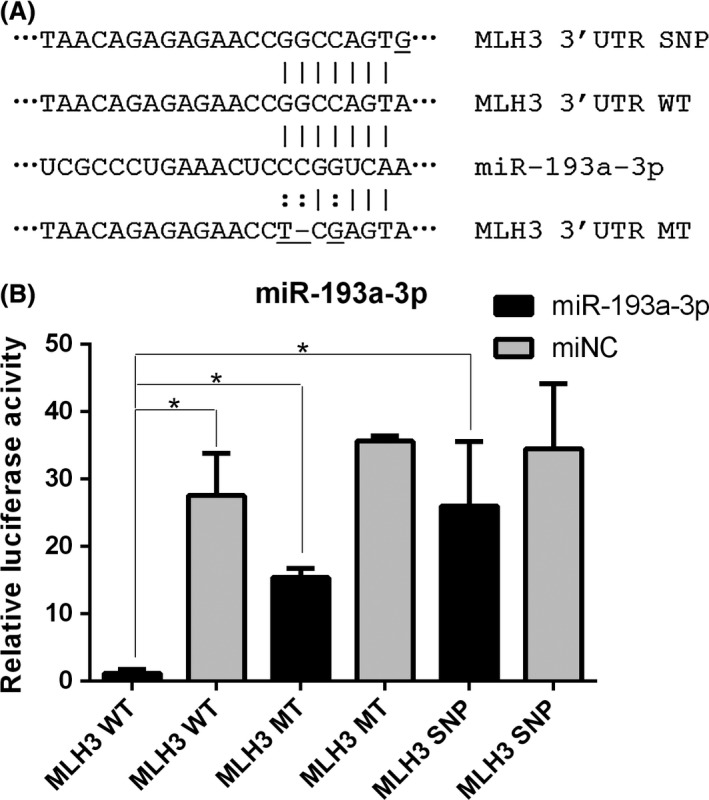
Luciferase reporter assay of miR‐193a‐3p. (A) miRNA binding sites in 3′UTR of MLH3 mRNA and mutations, SNP type. (B) Wild‐type, mutated‐type, or SNP‐type (SNP) reporter constructs were cotransfected into 293T cells with miR‐193a‐3p mimics or controls. The relative luciferase activities were measured (*P <0.05).

To investigate the possible interaction between miR‐193a‐3p and *MLH3*, and the effect of rs108621 on the regulation of miR‐193a to *MLH3*, luciferase reporter constructs generated with the wild‐type (WT), mutant type (MT), and SNP‐type (SNP) 3′ UTRs of *MLH3* were cotransfected into 293T cell with miR‐193a mimics or miRNA control (Fig 3A). Luciferase assays showed miR‐193a mimics decreased the expression of WT 3′UTR, and the expression level of MT and SNP 3′UTR was higher than WT 3′UTR with miR‐193a mimics existence (Fig 3B). The results reminded that miR‐193a‐3p directly regulated *MLH3* mRNA, and the SNP rs108621 G allele influenced the regulation.

**Figure 4:**
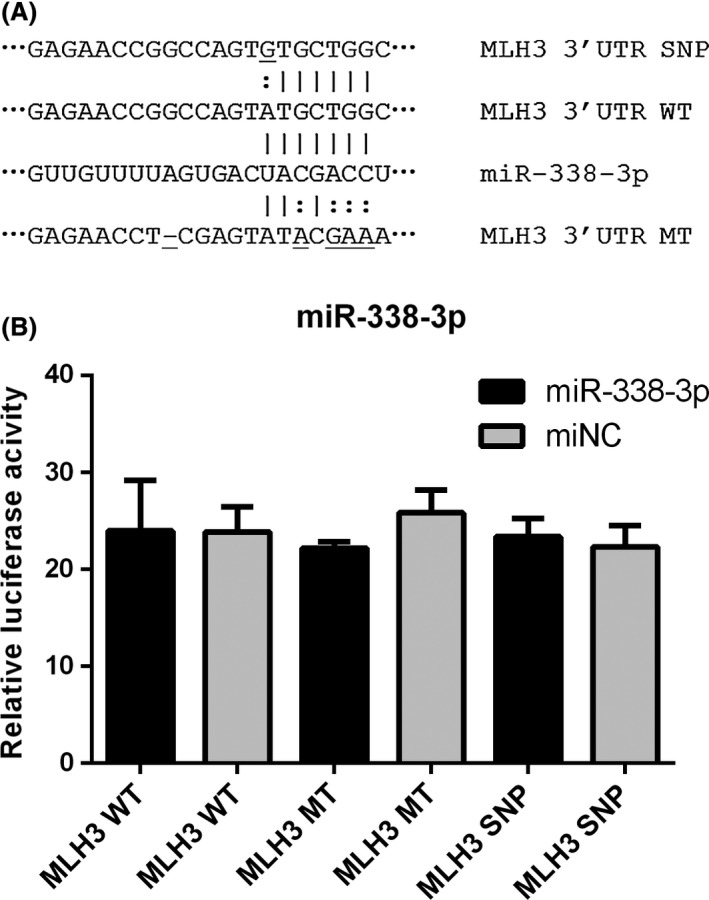
Luciferase reporter assay of miR‐338‐3p. (A) miRNA‐binding sites in 3′ UTR of MLH3 mRNA and the mutations, SNP type. (B) Wild‐type, mutated‐type, or SNP‐type (SNP) reporter constructs were cotransfected into 293T cells with miR‐338‐3p mimics or controls. The relative luciferase activities were measured.

Similarly, we analyzed the possible interaction between miR‐338‐3p and *MLH3* by luciferase reporter (Fig 4). But the data did not show any statistical difference.

## Discussion

Colorectal cancer is known as the malignant outcome of DNA repair system disorder, and at least three kinds of DNA repair pathways are reported to be involved in the development of CRC. For example, some inherited colon carcinomas were commonly reported to develop due to the deficient MMR, and DNA repair system which can correct for the mismatch of the base pairs repairs some nucleotide insertion or deletion shorter than 4nt. Generally speaking, six proteins (hMSH2, hMSH3, hMSH6, hMLH1, hMLH3, hPMS1, and hPMS2) participate in human MMR pathway 18. However, NER is a major DNA repair mechanism, which can contribute to the removal of bulky adducts and DNA cross‐links to maintain cellular genetic stability 19. BER pathway can handle DNA lesions that do not significantly distort the double helix. The process is mediated by multiple glycosylase and primarily repairs endogenous DNA damage such as deamination, alkylation, oxidation, and single‐strand breaks in genomic DNA 20. *ERCC1*,* MLH3,* and *OGG1* are the rate‐limiting DNA repair enzymes of three main DNA repair pathways known as NER, MMR, and BER. Therefore, our current study explored the possibility of the above DNA repair genes polymorphism as susceptibility biomarkers in predicting the risk of CRC.

Some SNPs of *ERCC1* were reported to have functions as valid biomarkers to predict CRC risk. *ERCC1* rs11615 C allele increased the risk of CRC in ever smokers and alcohol drinkers 21. A allele of *ERCC1* rs2336219 was reported to link with a higher CRC risk 22. Elevated *ERCC1* expression is significantly associated with unfavorable survival outcomes in patients with CRCs 23. In addition, *MLH3* participated in multiple biological processes such as DNA repair, microsatellite stability, and carcinogenesis 24. The AA genotype in *MLH3* rs175080 increased the risk of primary hepatocellular carcinoma for the Han population of northern China 25. The GA and AA genotypes of *MLH3* rs175080 were associated with a low risk of breast cancer 26. But till now, the association between *MLH3* polymorphisms and CRC risk was still unintelligible.

Although genomewide association studies (GWASs) and epidemiology investigation reminded the significance of polymorphisms in CDS, no enough data of SNPs on noncoding regions supported the above evidence, and the related mechanism was still uncertainty. As we have known, the variations located at 3′ UTR of DNA repair genes may affect gene expression due to its effect on post‐transcription regulation. In this study, *ERCC1* rs3212986 AA genotype increased the risk of CRC in male population. The rs3212986 polymorphism, located in the 3′‐untranslated region of *ERCC1* and the coding region of *CD3EAP,* may affect DNA repair capacity by reducing the stability of *ERCC1* mRNA 27. As we expected, a case–control study showed rs3212986 AA genotype was related with an increased risk of glioma 28. However, for the pancreatic cancer subjects with the CC genotype of rs3212986 compared with those with the AA genotype, no such significance was found 29. Therefore, rs3212986 was related with the risk of several cancers, but the conclusion is controversial. On the other hand, for *MLH3*, we found its rs108621 TT genotype was found to be related with a lower risk of CRC than CT + TT genotype in male, but interestingly, it showed an opposite result in female. This finding prompted that DNA repair gene may have gender differences. While *MLH3* rs108621 was related to CRC prognosis in 5‐FU treatment patients, patients carrying rs108621 CC genotype have a higher survival rate than the patients with CT + TT genotype 30.

Our current study showed both *MLH3* rs108621 C allele and *ERCC1* rs3212986 A allele were the harmful factors of CRC in male population. People carrying three “harmful alleles” had a higher risk of CRC than those who carrying less “harmful alleles.” Interestingly, there were 11 CRC cases (9.91%) carrying three “harmful alleles” and two patients (1.80%) carrying four “harmful alleles,” but in control group, there was no people carrying three or four “harmful alleles.” It seems to have a synergism of *MLH3* rs108621 C and *ERCC1* rs3212986 A alleles to predict the risk of CRC, which may provide a new and interesting data and contribute to predict the risk of CRC.

As we have known, polymorphisms in the miRNA‐binding region may be associated with the risk of CRC by altering the binding of miRNA–mRNA 31. Bioinformatics prediction reminded *ERCC1* rs3212986 was located at the predicted binding site of miR‐15a. It reminded that *ERCC1* could be regulated by miR‐15a via the binding site in the 3′UTR of the *ERCC1* mRNA, and *ERCC1* rs3212986 alters its binding affinity with miR‐15a. Similarly, an in vitro luciferase reporter assay was performed to confirm that *MLH3* rs108621 G allele decreased its binding with miR‐193a‐3p. In addition, as one of the potential blood biomarker, miR‐193a‐3p is used in early diagnosis of CRC 32. In a cohort of ulcerative colitis (UC) cancers, miR‐193a‐3p in cancer tissue was in a downregulation compared to its paracancer, and its lower expression promoted carcinogenesis through upregulation of *IL17RD* 33. Therefore, our data reminded miR‐193a‐3p may play a critical role in the association between *MLH3* rs108621 and the risk of CRC.

Although our current study reminded that rs108621 C allele together with rs3212986 A allele increased the risk of CRC especially in male population, and the influence of rs108621 to susceptibility of CRC may be mediated by miR‐193a‐3p, there were also some limitations in the present study. Such as a larger sample size and a more detailed functional study will be necessary to further validate the relation in the future.

## Conflict of Interest

None declared.

## Supporting information


**Table S1.** NER genes associated with CRC and relevance scoring.
**Table S2.** BER genes associated with CRC and relevance scoring.
**Table S3**. MMR genes associated with CRC and relevance scoring
**Table S4.** The prediction result of PolymiRTs.
**Figure S1**. The prediction result of Targetscan
