# Supplementary material for: The polymorphisms of miRNA‐binding site in MLH3 and ERCC1 were linked to the risk of colorectal cancer in a case–control study
Source: Cancer Med. 2018 Mar 8;7(4):1264–74. doi: 10.1002/cam4.1319 (PMC5911615; doi:10.1002/cam4.1319)
Supplement: Supplementary file 1 — Table S1. NER genes associated with CRC and relevance scoring. Table S2. BER genes associated with CRC and relevance scoring. Table S3. MMR genes associated with CRC and relevance scoring Table S4. The prediction result of PolymiRTs. Figure S1. The prediction result of Targetscan [file CAM4-7-1264-s001.docx]

Table S1, NER genes associated with CRC and relevance scoring

| Symbol | Description | [Score](http://www.genecards.org/Search/Keyword?queryString=colorectal+cancer+NER+Susceptibility&sort=Score&sortdir=ASC) |
| --- | --- | --- |
| *[EGFR](http://www.genecards.org/cgi-bin/carddisp.pl?gene=EGFR&keywords=colorectal,cancer,NER,Susceptibility)* | Epidermal Growth Factor Receptor | 61.40 |
| ***[ERCC1](http://www.genecards.org/cgi-bin/carddisp.pl?gene=ERCC1&keywords=colorectal,cancer,NER,Susceptibility)*** | **Excision Repair Cross-Complementation Group 1** | **37.63** |
| *[OGG1](http://www.genecards.org/cgi-bin/carddisp.pl?gene=OGG1&keywords=colorectal,cancer,NER,Susceptibility)* | 8-Oxoguanine DNA Glycosylase | 32.33 |
| *[PCNA](http://www.genecards.org/cgi-bin/carddisp.pl?gene=PCNA&keywords=colorectal,cancer,NER,Susceptibility)* | Proliferating Cell Nuclear Antigen | 30.64 |
| *[XRCC1](http://www.genecards.org/cgi-bin/carddisp.pl?gene=XRCC1&keywords=colorectal,cancer,NER,Susceptibility)* | X-Ray Repair Complementing Defective Repair In Chinese Hamster Cells 1 | 30.51 |
| *[ERCC2](http://www.genecards.org/cgi-bin/carddisp.pl?gene=ERCC2&keywords=colorectal,cancer,NER,Susceptibility)* | Excision Repair Cross-Complementation Group 2 | 28.17 |
| *[ATM](http://www.genecards.org/cgi-bin/carddisp.pl?gene=ATM&keywords=colorectal,cancer,NER,Susceptibility)* | ATM Serine/Threonine Kinase | 26.50 |
| *[NAT2](http://www.genecards.org/cgi-bin/carddisp.pl?gene=NAT2&keywords=colorectal,cancer,NER,Susceptibility)* | N-Acetyltransferase 2 (Arylamine N-Acetyltransferase) | 25.14 |
| *[EP300](http://www.genecards.org/cgi-bin/carddisp.pl?gene=EP300&keywords=colorectal,cancer,NER,Susceptibility)* | E1A Binding Protein P300 | 24.47 |
| *[POLD1](http://www.genecards.org/cgi-bin/carddisp.pl?gene=POLD1&keywords=colorectal,cancer,NER,Susceptibility)* | Polymerase (DNA Directed), Delta 1, Catalytic Subunit | 21.62 |
| *[POLE](http://www.genecards.org/cgi-bin/carddisp.pl?gene=POLE&keywords=colorectal,cancer,NER,Susceptibility)* | Polymerase (DNA Directed), Epsilon, Catalytic Subunit | 21.03 |
| *[PARP1](http://www.genecards.org/cgi-bin/carddisp.pl?gene=PARP1&keywords=colorectal,cancer,NER,Susceptibility)* | Poly (ADP-Ribose) Polymerase 1 | 18.68 |
| *[ERCC6](http://www.genecards.org/cgi-bin/carddisp.pl?gene=ERCC6&keywords=colorectal,cancer,NER,Susceptibility)* | Excision Repair Cross-Complementation Group 6 | 16.27 |
| *[XPC](http://www.genecards.org/cgi-bin/carddisp.pl?gene=XPC&keywords=colorectal,cancer,NER,Susceptibility)* | XerodermaPigmentosum, Complementation Group C | 16.17 |
| *[ERCC4](http://www.genecards.org/cgi-bin/carddisp.pl?gene=ERCC4&keywords=colorectal,cancer,NER,Susceptibility)* | Excision Repair Cross-Complementation Group 4 | 14.61 |
| *[ERCC5](http://www.genecards.org/cgi-bin/carddisp.pl?gene=ERCC5&keywords=colorectal,cancer,NER,Susceptibility)* | Excision Repair Cross-Complementation Group 5 | 14.04 |
| *[APEX1](http://www.genecards.org/cgi-bin/carddisp.pl?gene=APEX1&keywords=colorectal,cancer,NER,Susceptibility)* | APEX Nuclease (Multifunctional DNA Repair Enzyme) 1 | 12.72 |
| *[EXO1](http://www.genecards.org/cgi-bin/carddisp.pl?gene=EXO1&keywords=colorectal,cancer,NER,Susceptibility)* | Exonuclease 1 | 12.68 |
| *[NR1H2](http://www.genecards.org/cgi-bin/carddisp.pl?gene=NR1H2&keywords=colorectal,cancer,NER,Susceptibility)* | Nuclear Receptor Subfamily 1, Group H, Member 2 | 12.01 |
| *[ATR](http://www.genecards.org/cgi-bin/carddisp.pl?gene=ATR&keywords=colorectal,cancer,NER,Susceptibility)* | ATR Serine/Threonine Kinase | 10.23 |
| *[XPA](http://www.genecards.org/cgi-bin/carddisp.pl?gene=XPA&keywords=colorectal,cancer,NER,Susceptibility)* | XerodermaPigmentosum, Complementation Group A | 9.70 |
| *[ERCC3](http://www.genecards.org/cgi-bin/carddisp.pl?gene=ERCC3&keywords=colorectal,cancer,NER,Susceptibility)* | Excision Repair Cross-Complementation Group 3 | 9.05 |
| *[CREBBP](http://www.genecards.org/cgi-bin/carddisp.pl?gene=CREBBP&keywords=colorectal,cancer,NER,Susceptibility)* | CREB Binding Protein | 8.43 |
| *[POLB](http://www.genecards.org/cgi-bin/carddisp.pl?gene=POLB&keywords=colorectal,cancer,NER,Susceptibility)* | Polymerase (DNA Directed), Beta | 7.71 |
| *[RAD23B](http://www.genecards.org/cgi-bin/carddisp.pl?gene=RAD23B&keywords=colorectal,cancer,NER,Susceptibility)* | RAD23 Homolog B, Nucleotide Excision Repair Protein | 6.83 |

Table S2, BER genes associated with CRC and relevance scoring

| [Symbol](http://www.genecards.org/Search/Keyword?queryString=colorectal+cancer+BER+Susceptibility&sort=Symbol&sortdir=ASC) | Description | Score |
| --- | --- | --- |
| *[BCL2](http://www.genecards.org/cgi-bin/carddisp.pl?gene=BCL2&keywords=colorectal,cancer,BER,Susceptibility)* | B-Cell CLL/Lymphoma 2 | 43.73 |
| *[ESR1](http://www.genecards.org/cgi-bin/carddisp.pl?gene=ESR1&keywords=colorectal,cancer,BER,Susceptibility)* | Estrogen Receptor 1 | 40.00 |
| *[XRCC1](http://www.genecards.org/cgi-bin/carddisp.pl?gene=XRCC1&keywords=colorectal,cancer,BER,Susceptibility)* | X-Ray Repair Complementing Defective Repair In Chinese Hamster Cells 1 | 27.92 |
| *[PCNA](http://www.genecards.org/cgi-bin/carddisp.pl?gene=PCNA&keywords=colorectal,cancer,BER,Susceptibility)* | Proliferating Cell Nuclear Antigen | 27.89 |
| ***[OGG1](http://www.genecards.org/cgi-bin/carddisp.pl?gene=OGG1&keywords=colorectal,cancer,BER,Susceptibility)*** | **8-Oxoguanine DNA Glycosylase** | **26.78** |
| *[XRCC3](http://www.genecards.org/cgi-bin/carddisp.pl?gene=XRCC3&keywords=colorectal,cancer,BER,Susceptibility)* | X-Ray Repair Complementing Defective Repair In Chinese Hamster Cells 3 | 24.03 |
| *[PARP1](http://www.genecards.org/cgi-bin/carddisp.pl?gene=PARP1&keywords=colorectal,cancer,BER,Susceptibility)* | Poly (ADP-Ribose) Polymerase 1 | 23.29 |
| *[PGR](http://www.genecards.org/cgi-bin/carddisp.pl?gene=PGR&keywords=colorectal,cancer,BER,Susceptibility)* | Progesterone Receptor | 23.23 |
| *[KRT7](http://www.genecards.org/cgi-bin/carddisp.pl?gene=KRT7&keywords=colorectal,cancer,BER,Susceptibility)* | Keratin 7, Type II | 22.69 |
| *[CEACAM5](http://www.genecards.org/cgi-bin/carddisp.pl?gene=CEACAM5&keywords=colorectal,cancer,BER,Susceptibility)* | Carcinoembryonic Antigen-Related Cell Adhesion Molecule 5 | 21.95 |
| *[MUC1](http://www.genecards.org/cgi-bin/carddisp.pl?gene=MUC1&keywords=colorectal,cancer,BER,Susceptibility)* | Mucin 1, Cell Surface Associated | 20.95 |
| *[KRT19](http://www.genecards.org/cgi-bin/carddisp.pl?gene=KRT19&keywords=colorectal,cancer,BER,Susceptibility)* | Keratin 19, Type I | 18.04 |
| *[MKI67](http://www.genecards.org/cgi-bin/carddisp.pl?gene=MKI67&keywords=colorectal,cancer,BER,Susceptibility)* | Marker Of Proliferation Ki-67 | 16.85 |
| *[CEACAM3](http://www.genecards.org/cgi-bin/carddisp.pl?gene=CEACAM3&keywords=colorectal,cancer,BER,Susceptibility)* | Carcinoembryonic Antigen-Related Cell Adhesion Molecule 3 | 14.81 |
| *[APEX1](http://www.genecards.org/cgi-bin/carddisp.pl?gene=APEX1&keywords=colorectal,cancer,BER,Susceptibility)* | APEX Nuclease (Multifunctional DNA Repair Enzyme) 1 | 14.15 |
| *[XPC](http://www.genecards.org/cgi-bin/carddisp.pl?gene=XPC&keywords=colorectal,cancer,BER,Susceptibility)* | XerodermaPigmentosum, Complementation Group C | 10.27 |
| *[THBD](http://www.genecards.org/cgi-bin/carddisp.pl?gene=THBD&keywords=colorectal,cancer,BER,Susceptibility)* | Thrombomodulin | 8.72 |
| *[PON1](http://www.genecards.org/cgi-bin/carddisp.pl?gene=PON1&keywords=colorectal,cancer,BER,Susceptibility)* | Paraoxonase 1 | 8.19 |
| *[UNG](http://www.genecards.org/cgi-bin/carddisp.pl?gene=UNG&keywords=colorectal,cancer,BER,Susceptibility)* | Uracil DNA Glycosylase | 7.12 |
| *[LIG3](http://www.genecards.org/cgi-bin/carddisp.pl?gene=LIG3&keywords=colorectal,cancer,BER,Susceptibility)* | Ligase III, DNA, ATP-Dependent | 6.18 |
| *[CALB2](http://www.genecards.org/cgi-bin/carddisp.pl?gene=CALB2&keywords=colorectal,cancer,BER,Susceptibility)* | Calbindin 2 | 5.96 |
| *[CD34](http://www.genecards.org/cgi-bin/carddisp.pl?gene=CD34&keywords=colorectal,cancer,BER,Susceptibility)* | CD34 Molecule | 5.67 |
| *[HMGB1](http://www.genecards.org/cgi-bin/carddisp.pl?gene=HMGB1&keywords=colorectal,cancer,BER,Susceptibility)* | High Mobility Group Box 1 | 5.20 |
| *[TNFRSF8](http://www.genecards.org/cgi-bin/carddisp.pl?gene=TNFRSF8&keywords=colorectal,cancer,BER,Susceptibility)* | Tumor Necrosis Factor Receptor Superfamily, Member 8 | 4.14 |
| *[FEN1](http://www.genecards.org/cgi-bin/carddisp.pl?gene=FEN1&keywords=colorectal,cancer,BER,Susceptibility)* | Flap Structure-Specific Endonuclease 1 | 3.97 |

Table S3, MMR genes associated with CRC and relevance scoring

| Symbol | Description | [Score](http://www.genecards.org/Search/Keyword?queryString=colorectal+cancer+MMR+Susceptibility&sort=Score&sortdir=ASC) |
| --- | --- | --- |
| *[MLH1](http://www.genecards.org/cgi-bin/carddisp.pl?gene=MLH1&keywords=colorectal,cancer,MMR,Susceptibility)* | MutL Homolog 1 | 87.35 |
| *[TP53](http://www.genecards.org/cgi-bin/carddisp.pl?gene=TP53&keywords=colorectal,cancer,MMR,Susceptibility)* | Tumor Protein P53 | 85.45 |
| *[MSH2](http://www.genecards.org/cgi-bin/carddisp.pl?gene=MSH2&keywords=colorectal,cancer,MMR,Susceptibility)* | MutS Homolog 2 | 76.59 |
| *[MSH6](http://www.genecards.org/cgi-bin/carddisp.pl?gene=MSH6&keywords=colorectal,cancer,MMR,Susceptibility)* | MutS Homolog 6 | 62.35 |
| *[CTNNB1](http://www.genecards.org/cgi-bin/carddisp.pl?gene=CTNNB1&keywords=colorectal,cancer,MMR,Susceptibility)* | Catenin (Cadherin-Associated Protein), Beta 1, 88kDa | 57.20 |
| *[KRAS](http://www.genecards.org/cgi-bin/carddisp.pl?gene=KRAS&keywords=colorectal,cancer,MMR,Susceptibility)* | Kirsten Rat Sarcoma Viral Oncogene Homolog | 56.99 |
| *[BRCA1](http://www.genecards.org/cgi-bin/carddisp.pl?gene=BRCA1&keywords=colorectal,cancer,MMR,Susceptibility)* | Breast Cancer 1, Early Onset | 54.73 |
| *[CDH1](http://www.genecards.org/cgi-bin/carddisp.pl?gene=CDH1&keywords=colorectal,cancer,MMR,Susceptibility)* | Cadherin 1, Type 1 | 53.38 |
| *[PTEN](http://www.genecards.org/cgi-bin/carddisp.pl?gene=PTEN&keywords=colorectal,cancer,MMR,Susceptibility)* | Phosphatase And Tensin Homolog | 49.53 |
| *[IL6](http://www.genecards.org/cgi-bin/carddisp.pl?gene=IL6&keywords=colorectal,cancer,MMR,Susceptibility)* | Interleukin 6 | 47.46 |
| *[BRCA2](http://www.genecards.org/cgi-bin/carddisp.pl?gene=BRCA2&keywords=colorectal,cancer,MMR,Susceptibility)* | Breast Cancer 2, Early Onset | 47.22 |
| *[APC](http://www.genecards.org/cgi-bin/carddisp.pl?gene=APC&keywords=colorectal,cancer,MMR,Susceptibility)* | Adenomatous Polyposis Coli | 46.15 |
| *[BRAF](http://www.genecards.org/cgi-bin/carddisp.pl?gene=BRAF&keywords=colorectal,cancer,MMR,Susceptibility)* | B-Raf Proto-Oncogene, Serine/Threonine Kinase | 45.94 |
| *[CDKN2A](http://www.genecards.org/cgi-bin/carddisp.pl?gene=CDKN2A&keywords=colorectal,cancer,MMR,Susceptibility)* | Cyclin-Dependent Kinase Inhibitor 2A | 45.53 |
| *[PMS2](http://www.genecards.org/cgi-bin/carddisp.pl?gene=PMS2&keywords=colorectal,cancer,MMR,Susceptibility)* | PMS1 Homolog 2, Mismatch Repair System Component | 44.52 |
| *[IFNG](http://www.genecards.org/cgi-bin/carddisp.pl?gene=IFNG&keywords=colorectal,cancer,MMR,Susceptibility)* | Interferon, Gamma | 32.50 |
| *[CDKN1A](http://www.genecards.org/cgi-bin/carddisp.pl?gene=CDKN1A&keywords=colorectal,cancer,MMR,Susceptibility)* | Cyclin-Dependent Kinase Inhibitor 1A (P21, Cip1) | 31.03 |
| *[MUTYH](http://www.genecards.org/cgi-bin/carddisp.pl?gene=MUTYH&keywords=colorectal,cancer,MMR,Susceptibility)* | MutY DNA Glycosylase | 29.72 |
| *[PCNA](http://www.genecards.org/cgi-bin/carddisp.pl?gene=PCNA&keywords=colorectal,cancer,MMR,Susceptibility)* | Proliferating Cell Nuclear Antigen | 29.03 |
| *[FASLG](http://www.genecards.org/cgi-bin/carddisp.pl?gene=FASLG&keywords=colorectal,cancer,MMR,Susceptibility)* | Fas Ligand (TNF Superfamily, Member 6) | 26.76 |
| *[PMS1](http://www.genecards.org/cgi-bin/carddisp.pl?gene=PMS1&keywords=colorectal,cancer,MMR,Susceptibility)* | PMS1 Homolog 1, Mismatch Repair System Component | 26.61 |
| ***[MLH3](http://www.genecards.org/cgi-bin/carddisp.pl?gene=MLH3&keywords=colorectal,cancer,MMR,Susceptibility)*** | **MutL Homolog 3** | **26.08** |
| *[MSH3](http://www.genecards.org/cgi-bin/carddisp.pl?gene=MSH3&keywords=colorectal,cancer,MMR,Susceptibility)* | MutSHomolog 3 | 25.71 |
| *[MGMT](http://www.genecards.org/cgi-bin/carddisp.pl?gene=MGMT&keywords=colorectal,cancer,MMR,Susceptibility)* | O-6-Methylguanine-DNA Methyltransferase | 24.18 |
| *[IL2](http://www.genecards.org/cgi-bin/carddisp.pl?gene=IL2&keywords=colorectal,cancer,MMR,Susceptibility)* | Interleukin 2 | 23.26 |


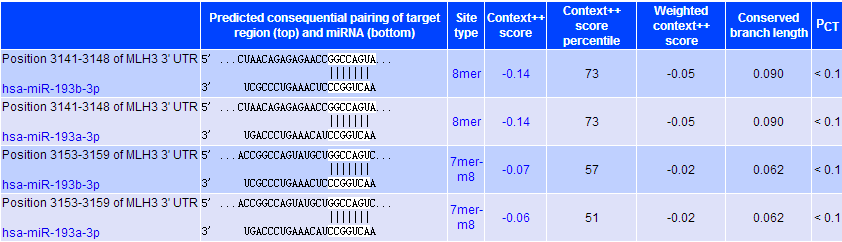


Figure s1.The prediction result of Targetscan.

Table s4.The prediction result of PolymiRTs

| dbSNP ID | Variant type | Wobble base pair | Ancestral Allele | Allele | miR ID | Conservation | miRSite | Function Class | Exp Support | context+ score change |
| --- | --- | --- | --- | --- | --- | --- | --- | --- | --- | --- |
| [rs108621](http://www.ncbi.nlm.nih.gov/SNP/snp_ref.cgi?rs=rs108621) | SNP | Y | A | A | [hsa-miR-193a-3p](http://www.mirbase.org/cgi-bin/query.pl?terms=hsa-miR-193a-3p)  [hsa-miR-193b-3p](http://www.mirbase.org/cgi-bin/query.pl?terms=hsa-miR-193b-3p)  [hsa-miR-338-3p](http://www.mirbase.org/cgi-bin/query.pl?terms=hsa-miR-338-3p)  [hsa-miR-892b](http://www.mirbase.org/cgi-bin/query.pl?terms=hsa-miR-892b) | [2](http://compbio.uthsc.edu/miRSNP/miRSNP_detail_all.php) [2](http://compbio.uthsc.edu/miRSNP/miRSNP_detail_all.php) [2](http://compbio.uthsc.edu/miRSNP/miRSNP_detail_all.php) [2](http://compbio.uthsc.edu/miRSNP/miRSNP_detail_all.php) | GCCAGTAtgctgg GCCAGTAtgctgg gccagtATGCTGG GCCAGTAtgctgg | D D D D | N N N N | -0.221 -0.23 -0.119 -0.151 |
|  |  |  |  | G | [hsa-miR-6757-3p](http://www.mirbase.org/cgi-bin/query.pl?terms=hsa-miR-6757-3p)  [hsa-miR-8064](http://www.mirbase.org/cgi-bin/query.pl?terms=hsa-miR-8064) | [3](http://compbio.uthsc.edu/miRSNP/miRSNP_detail_all.php) [2](http://compbio.uthsc.edu/miRSNP/miRSNP_detail_all.php) | gCCAGTGTgctgg gccAGTGTGCtgg | C C | N N | -0.075 -0.079 |
